# Understanding Anthropogenic PM_2.5_ Concentrations and Their Drivers in China during 1998–2016

**DOI:** 10.3390/ijerph20010695

**Published:** 2022-12-30

**Authors:** Guoliang Yun, Chen Yang, Shidong Ge

**Affiliations:** 1 College of Landscape Architecture and Art, Henan Agricultural University, Zhengzhou 450002, China; 2 College of Urban and Environmental Sciences, and Key Laboratory for Earth Surface Processes, Ministry of Education, Peking University, Beijing 100871, China

**Keywords:** anthropogenic PM_2.5_ concentrations, socioeconomic factors, trend analysis, grey correlation analysis

## Abstract

Air pollution poses serious challenges for human health and wellbeing. It also affects atmospheric visibility and contributes to climate change. As social and economic processes have increased, anthropogenic PM_2.5_ pollution caused by intensive human activities has led to extremely severe air pollution. Spatiotemporal patterns and drivers of anthropogenic PM_2.5_ concentrations have received increasing attention from the scientific community. Nonetheless, spatiotemporal patterns and drivers of anthropogenic PM_2.5_ concentrations are still inadequately understood. Based on a time series of remotely sensed anthropogenic PM_2.5_ concentrations, this study analyzed the spatiotemporal patterns of this crucial pollutant in China from 1998 to 2016 using Sen’s slope estimator and the Mann–Kendall trend model. This, in combination with grey correlation analysis (GCA), was used to reveal the socioeconomic factors influencing anthropogenic PM_2.5_ concentrations in eastern, central, and western China from 1998 to 2016. The results were as follows: (1) the average annual anthropogenic concentration of PM_2.5_ in China increased quickly and reached its peak value in 2007, then remained stable in the following years; (2) only 63.30 to 55.09% of the land area reached the threshold value of 15 μg/m^3^ from 1998 to 2016; (3) regarding the polarization phenomenon of anthropogenic PM_2.5_ concentrations existing in eastern and central China, the proportion of gradient 1 (≤15 μg/m^3^) gradually decreased and gradient 3 (≥35 μg/m^3^) gradually increased; and (4) the urbanization level (UR), population density (PD), and proportion of secondary industry to gross domestic product (SI) were the dominant socioeconomic factors affecting the formation of anthropogenic PM_2.5_ concentrations in eastern, central, and western China, independently. The improvements in energy consumption per gross domestic product (EI) have a greater potential for mitigating anthropogenic PM_2.5_ emissions in central and western China. These findings allow an interpretation of the spatial distribution of anthropogenic PM_2.5_ concentrations and the mechanisms influencing anthropogenic PM_2.5_ concentrations, which can help the Chinese government develop effective abatement strategies.

## 1. Introduction

Rapid urbanization has received much attention in recent years due to its sobering impact on social, economic, and environmental dimensions [1,2,3]. More and more empirical analysis of domestic and international research has shown that air pollutant emissions have increased dramatically with rapid urbanization [4,5,6,7]. As a primary air pollutant, PM_2.5_ is a complex mixture of anthropogenic and natural sources including sulfate, nitrate, ammonium, carbonaceous aerosols, sand dust, and sea salt. With its small particle size, PM_2.5_ also easily adheres to toxic substances such as persistent organic pollutants, heavy metals, and pathogenic bacteria [8,9,10]. As a result, inhaled PM_2.5_ can lead to cardiovascular disease, heart disease, and respiratory diseases [11,12,13,14]. Anthropogenic PM_2.5_ concentrations contribute substantially to global premature mortality [15,16,17]. More than 80% of global PM_2.5_-attributable deaths were related to anthropogenic sources [18]. Anthropogenic PM_2.5_ pollution was associated with 3.5 ± 0.9 million cardiopulmonary and 220,000 ± 80,000 lung cancer mortalities (30 ± 7.6 million years of life lost) annually [19,20]. More than half of these deaths occurred in areas undergoing rapid industrialization and urbanization in East Asia and South Asia [21]. In Africa, anthropogenic PM_2.5_ pollution contributes to 13,210 premature deaths annually [22]. Therefore, understanding the spatiotemporal patterns and drivers of anthropogenic PM_2.5_ pollution is crucially important in making decisions to develop air quality management strategies and protect public health.

Over the past few years, a growing body of studies have examined the spatial and temporal distribution patterns and trends of PM_2.5_ concentrations on different scales, from global, continent, and country to regional levels. On a global scale, [13] first inverted the global distribution of PM_2.5_ concentrations using the atmospheric chemical transport model (GEOS-Chem) in combination with MODIS AOD and MISR AOD products and found that the highest PM_2.5_ concentrations were located in eastern China and northern India. After this, Hammer et al. [23] further improved the accuracy of annual mean PM_2.5_ concentrations and the time length of the data using advances in satellite observations, chemical transport modeling, and ground-based monitoring. On a continent scale, Lijian et al. [24] analyzed PM_2.5_ concentrations on each continent based on the global PM_2.5_ concentrations’ dataset spatial resolution of 10 km and found that North America, Europe, and Latin America have better air quality than Asia and Africa. On a national and regional scale, the study of Luo et al. [25] analyzed the spatial and temporal patterns of PM_2.5_ concentrations in mainland China and eight regions and found that areas that exceeded 35 μg/m^3^ expanded from the central eastern region to the southwestern region during the period of 1998–2012. However, these studies mainly focused on all components that are included when measuring PM_2.5_ concentrations in order to analyze the spatial distribution and temporal variations in PM_2.5_ pollution and did not distinguish between the sources [26,27].

Some studies have been conducted regarding changes in anthropogenic PM_2.5_ concentrations and their causes, and research in this area is ongoing [28,29,30]. For example, Crouse et al. [31] analyzed the changes in anthropogenic PM_2.5_ concentrations in southern Canada based on the AOD and GEOS-chem model. Sakunkoo et al. [17] analyzed the spatial variation in anthropogenic PM_2.5_ concentrations in Khon Kaen province, Thailand, between December 2020 and February 2021. Querol et al. [32] investigated the change in anthropogenic PM_2.5_ concentrations in the Castelló province in July 1999. In terms of the drivers of anthropogenic PM_2.5_ concentrations, Lim et al. [30] investigated the relationship between population, urbanization levels, vegetation greenness, and concentrations of anthropogenic PM_2.5_. Yue et al. [29] studied the relationship between anthropogenic PM_2.5_ concentration changes and income per capita in four groups with different income levels. However, most studies mainly focused on short-term impacts at a city level or the scale of provinces regarding the effects of anthropogenic PM_2.5_ pollution; studies have not looked at this pollutant on a regional or national scale. In addition, although it is clear that multiple socioeconomic factors contribute to haze pollution, few studies have comprehensively investigated the relationship between socioeconomic factors and anthropogenic PM_2.5_ concentrations in terms of urbanization levels, population density, per capita income, industrial organization, and energy intensity. Consequently, analysis of the spatiotemporal patterns and driving forces of anthropogenic PM_2.5_ pollution has not yet been adequately conducted so that the impact of this pollutant can be fully understood and addressed.

China’s rapid economic growth has been accompanied by rapid industrialization and urbanization since the “Reform and Opening-up” policy established in 1978 [26,33]. The urban population proportion in China has increased from 17.9% in 1978 to 60.6% in 2019 [34], and it is projected that 65% or approximately one billion Chinese people will live in cities by 2030 [3,35]. This growth, in such a short period, has not only brought great wealth to China and improved the living standards of the residents but has caused severe air pollution and remarkably modified the spatial distribution of PM_2.5_ emissions as well [10,36,37,38]. In response, in this study, we attempt to investigate the spatiotemporal trends of anthropogenic PM_2.5_ concentrations across space and time based on Sen’s slope estimator and the Mann–Kendall trend model in China from 1998 to 2016. In addition, the driving force analysis, in combination with grey correlation analysis (GCA), was used to reveal the socioeconomic factors influencing anthropogenic PM_2.5_ concentrations. The goals of this work include addressing the following research questions: (1) what were the spatial patterns, temporal dynamics, and levels of anthropogenic PM_2.5_ concentrations in China from 1998 to 2016?; (2) what are the driving forces of change in the anthropogenic PM_2.5_ emissions in China?; and (3) which measures should we take to mitigate anthropogenic PM_2.5_ emissions and improve air quality in China?

## 2. Materials and Methods

### 2.1. Anthropogenic PM_2.5_ Data

This study used annual average anthropogenic PM_2.5_ data obtained from the Atmospheric Composition Analysis Group [6,39]. The anthropogenic PM_2.5_ data were based on a simulation of the GEOS-Chem chemical transport model, and the PM_2.5_ concentrations were estimated from a combination of multiple satellite products (MISR, MODIS Dark Target, MODIS, and SeaWiFS Deep Blue) with aerosol vertical profiles and scattering properties [13]. The spatial resolution was 0.1° × 0.1°.

Anthropogenic PM_2.5_ concentration data exclude natural dust and sea salt [6,40,41]. A detailed description and verification of the dataset were provided by [6] globally. The correlation analysis shows the dataset highly corresponded with monitoring stations data in China and India (R = 0.81), which indicated that the accuracy of this dataset could support analysis [29]. A detailed description and verification of the dataset were provided by [29] for China and India. The resulting anthropogenic PM_2.5_ data were used for this study. In this study, we also used a subset of the global anthropogenic PM_2.5_ data that covered China, and the temporal range was from 1998 to 2016.

### 2.2. Socioeconomic Factors

The spatial variation in anthropogenic PM_2.5_ concentrations is significant. The reasons for this variation are complex, and the driving forces are diverse [42]. Previous studies have found that anthropogenic PM_2.5_ concentrations are significantly affected by socioeconomic factors such as urbanization levels [43], population density [44], gross domestic product [45], energy intensity [46], and industrial structures [47]. Based on previous research and considering data availability, we selected the following five impact factors for analysis: (1) urbanization level (UR) (urban population divided by total population, which refers to the physical growth of urban areas due to the migration of people from rural areas to urban or industrial areas); (2) population density (PD), measured as population per unit area, to indicate the impact of population agglomeration on anthropogenic PM_2.5_ pollution; (3) per capita gross domestic product (PGDP), as an indicator for measuring economic development and standards of living; (4) energy consumption per gross domestic product (EI), which illustrates the extent of energy use in a region’s economic activities, reflecting changes in economic structure and energy efficiency; and (5) the proportion of secondary industry to gross domestic product (SI), which reflects changes in economic structure and industrialization. The UR, PD, PGDP, EI, and SI values were obtained from the National Bureau of Statistics of the People’s Republic of China (http://www.stats.gov.cn/tjsj/, accessed on 10 December 2019).

**Figure 1:**
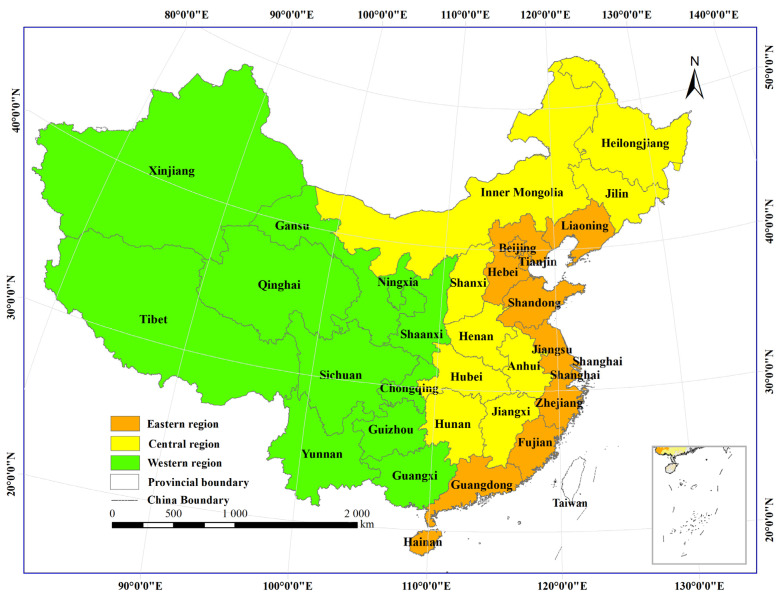
The locations of the three regions in China; the highlighted square in the right corner is “South China Sea territory map”.

**Figure 2:**
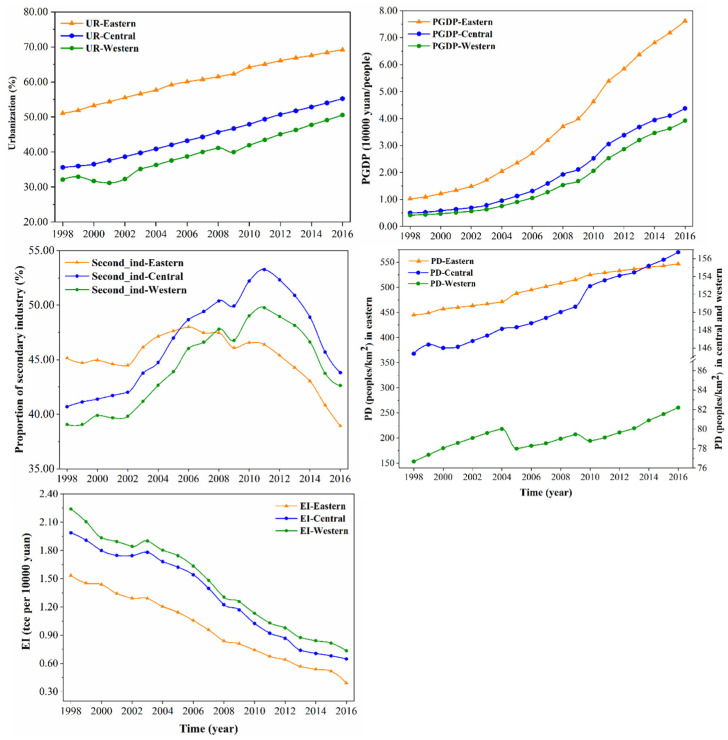
UR, PGDP, SI, PD, and EI in the three regions of China during 1998–2016.

In this study, mainland China is divided into three regions according to the division of the Seventh 5-Year Plan for National Economic and Social Development of the People’s Republic of China, issued in 1985 [48,49,50]. The specific classification results are shown in Figure 1. The statistics of impact factors for China and the three regions are summarized in Figure 2 and Table 1 and Table 2.

### 2.3. Analysis Models

#### 2.3.1. Sen’s Slope Estimator

The Sen’s slope estimator method was proposed by Sen in 1968 and is a non-parametric procedure for estimating the slope of the trend in a sample [51]. The core of the method is to construct the order sequence of the sample sequence at different change rates. The statistical variable (Sen’s slope) test is performed according to the given significance level, and the range of the change rate is obtained. Finally, the trend and magnitude of the sequence are judged by the median size. For a set of time series, $X_{i}=\left(x_{1},x_{2},\ldots ,x_{n}\right)$; the slope denoting the direction and quantity of the change trend can be written as:

(1) $$Q_{ij}=median\frac{\left(x_{i}-x_{j}\right)}{i-j},\;1\;<j<i<n$$

where $x_{i}$ and $x_{j}$ are the values of the data at times i and j (i>j), respectively. When there is only one set of time series, $N=\frac{n\left(n-1\right)}{2}$; when there are multiple sets of time series, $N<\frac{n\left(n-1\right)}{2}$, where n is the total number of observations. In the sequence *X*, the N values of $Q_{i}$ are arranged from small to large. The $Q_{median}$ of the slope is computed as:

(2) $$Q=\{\begin{array}{c} Q_{\left[\frac{\left(n+1\right)}{2}\right]},\;\;\;\;\;\;\;\;\;\;\;\;\;\;\;\;\;\;\;\;\;\;\;\;\;\;\;\;\mathrm{if}\;N\;\mathrm{is}\;\mathrm{odd}\\ \frac{Q_{\left[\frac{\left(n\right)}{2}\right]}+Q_{\left[\frac{\left(n+2\right)}{2}\right]}}{2},\;\;\;\;\;\;\;\;\;\;\;\;\;\;\mathrm{if}\;N\;\mathrm{is}\;\mathrm{even} \end{array}$$

The absolute value of Sen’s slope represents the magnitude of the sequence change; if Sen’s slope >0, the sequence shows an increasing trend, whereas if Sen’s slope <0, the sequence shows a decreasing trend. Sen’s slope method can reduce or avoid the influence of data anomalies and missing data on the analysis results when evaluating the trend of a time series and the magnitude of the change (the rate of change). It is also a mature statistical method for analyzing the magnitude of changes in hydrometeorological systems. In recent years, this method has also been applied to the trend analysis of hydrological and remote sensing time series data [52,53]. We used the Mann–Kendall method to identify whether Sen’s slope trend is significant.

#### 2.3.2. Mann–Kendall Trend Model

The Mann–Kendall trend model is a non-parametric procedure for determining the changing trend of time series data [54]:

(3) $$S=\overset{n-1}{\underset{i=1}{\sum }}\overset{n}{\underset{j=i+1}{\sum }}sign\left(x_{j}-x_{i}\right)$$

(4) $$sign\left(x_{j}-x_{i}\right)=\{\begin{array}{c} 1,x_{j}-x_{i}>0\\ 0,x_{j}-x_{i}=0\\ -1,x_{j}-x_{i}<0 \end{array}$$

(5) $$S_{t}=\left[n\left(n-1\right)\left(2n-5\right)-\overset{q}{\underset{p=1}{\sum }}t_{p}\left(t_{p}-1\right)\left(2t_{p}+5\right)\right]/18$$

(6) $$Z=\{\begin{array}{c} \frac{S-1}{\sqrt{S_{t}}}\;\;\;S_{t}>0\\ \;\;0\;\;\;\;\;\;\;S_{t}=0\\ \frac{S+1}{\sqrt{S_{t}}}\;\;S_{t}<0 \end{array}$$

where *Z* > 0 stands for an increasing trend, and *Z* < 0 stands for a decreasing trend. The absolute values of *Z* are greater than or equal to 1.96, indicating reliability of 95% and 99%, respectively. Thus, $H_{0}$ should be accepted where *|Z|* ≤ $z_{1-\alpha /2}$ in a two-sided test for trend at significant level α. *H*_0_ refers to Hypothesis 0 and indicates that there is no change in the trend for the anthropogenic PM_2.5_ concentration sequence. Hypothesis 1 (*H*_1_) indicates that the anthropogenic PM_2.5_ concentration sequence presents an increasing or decreasing trend.

The resulting trend using this method was divided into four grades, where trend <0 indicated decrease, 0 < trend < 0.5 a slight increase, 0.5 ≤ trend < 1 a moderated increase, and trend ≥ 1 a severe increase.

#### 2.3.3. Grey Correlation Analysis

Grey correlation analysis (GCA) is a method proposed by [55] to measure the similarity between reference factors and influencing factors through correlation degree based on system engineering. The core of the method is to determine the main influencing factors by calculating the degree of similarity between the reference sequence and the geometry of the compared sequence, which corresponds to the degree of association.

Due to its simplicity of use, simple structure, and wide application range, GCA has been widely used in the field of larger scale pollution analysis [56,57,58]. The steps of GCA are as follows: First, determine the target sequence and the compare sequence: the target sequence $X_{0}=\left\{x_{0}\left(k\right),k=1,2,\ldots ,N\right\}$ and the compared sequence $X_{i}=\left\{x_{i}\left(k\right),k=1,2,\ldots ,N,i=1,2,\ldots ,m\right\}.$ Second, standardize the data. This study used the mean method to standardize the dimensional differences of the original indicator data. The mean method is computed as:

(7) $$x_{j}\left(k\right)=\frac{x_{j}\left(k\right)}{\underset{i}{\max}x_{j}\left(k\right)}\left(j=1,2,\ldots ,m\right)$$

where $\max x_{j}\left(k\right)$ is the maximum for the *j*th sequence.

Third, calculate the grey correlation coefficient. This correlation coefficient is expressed as:

(8) $$\xi_{i}\left(k\right)=\frac{\underset{}{\min_{i}}\underset{}{\min_{k}}\left|x_{0}\left(k\right)-x_{i}\left(k\right)\right|+\rho *\underset{}{\max_{i}}\underset{}{\max_{k}}\left|x_{0}\left(k\right)-x_{i}\left(k\right)\right|}{\left|x_{0}\left(k\right)-x_{i}\left(k\right)\right|+\rho *\underset{}{\max_{i}}\underset{}{\max_{k}}\left|x_{0}\left(k\right)-x_{i}\left(k\right)\right|}$$

where ρ is the resolution’s coefficient.

Finally, calculate the grey correlation degrees as:

(9) $$r_{i}=\frac{1}{N}\sum_{k=1}^{N}\xi_{i}\left(k\right)\;\left(i=1,2,\ldots ,m\right)$$

where $r_{i}$ is the grey correlation between the target and compare sequence. The resolution coefficient, ρ, is a key parameter for the calculation of the grey correlation; normally, ρ is 0.5, and therefore, in this study, ρ was set to 0.5 (Zhu et al., 2018 [59]). The value of $r_{i}$ ranges from 0 to 1. Generally, if 0 < $r_{i}$ ≤ 0.30, the correlation is considered to be low; if 0.30 < $r_{i}$ ≤ 0.60, the correlation is moderate; if 0.60 < $r_{i}$ ≤ 1.0, the correlation is strong.

## 3. Results

### 3.1. Spatial Distribution and Temporal Variations in Anthropogenic PM_2.5_ Concentrations

**Figure 3:**
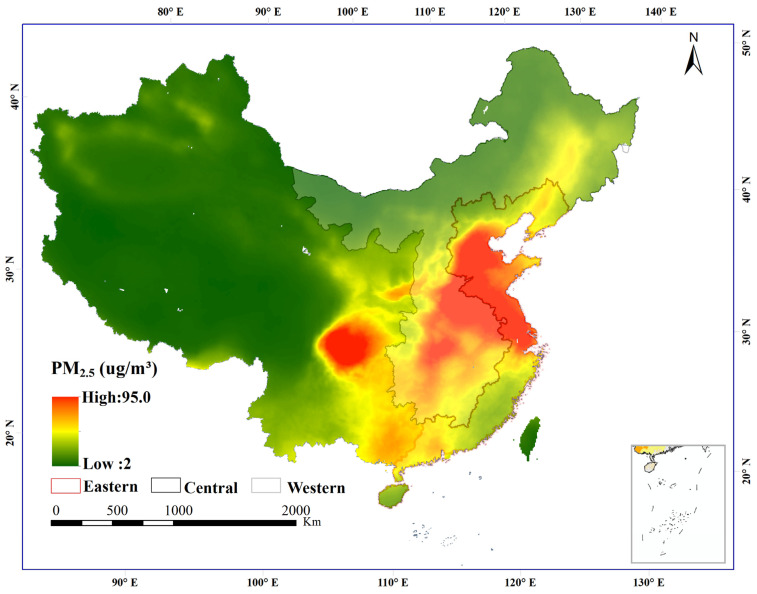
The spatial distribution of the annual average anthropogenic PM_2.5_ concentrations in China during 1998–2016. (We used ArcGIS 10.2 software to calculate the average mean of the gridded data of anthropogenic PM_2.5_ concentrations from 1998 to 2016).

Figure 3 demonstrates that annual anthropogenic PM_2.5_ concentrations were high in central and eastern China, low in the western region, and decreased from the southeast to the northwest. Specifically, the high anthropogenic PM_2.5_ concentrations occurred over the Beijing-Tianjin-Hebei urban agglomeration (BTH), Henan province, the Yangtze River Delta urban agglomeration (YRD), the Pearl River Delta urban agglomeration (PRD), and the Chongqing-Chengdu urban agglomeration (CHC). The areas with moderate anthropogenic PM_2.5_ concentrations occurred in the Harbin-Changchun urban agglomeration (HC), Hunan province, and Jiangxi province. The low anthropogenic PM_2.5_ concentrations zone was located in the Qinghai-Xizang Plateau in northeast China, Gansu province, and the Inner Mongolia autonomous region.

**Figure 4:**
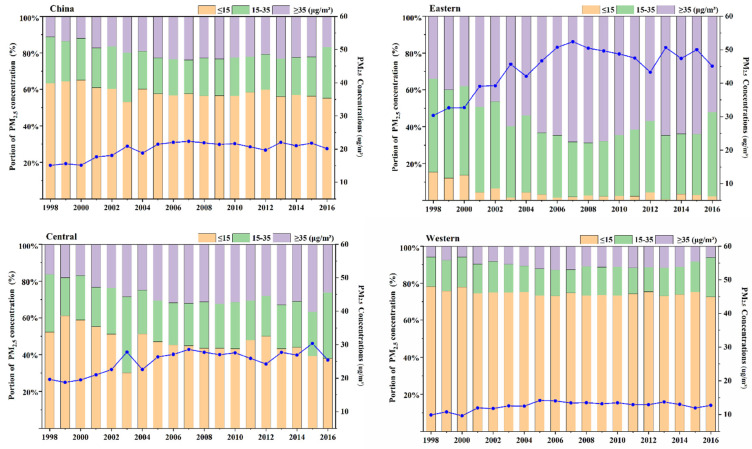
Changes in anthropogenic PM_2.5_ concentrations by range in China during 1998–2016 (the blue line represents the average annual anthropogenic PM_2.5_ concentrations of regions during 1998–2016).

In terms of PM_2.5_ changes over time, the change in annual average anthropogenic PM_2.5_ concentrations showed two phases, namely, a rapid upward trend from 1998 to 2007 and a stable trend from 2008 onwards. From 1998 to 2007, the annual average anthropogenic PM_2.5_ concentrations showed a continuous upward trend, with the annual average anthropogenic PM_2.5_ concentrations increasing from 15.10 $\mu g/m^{3}$ in 1998 to 22.34 $\mu g/m^{3}$ in 2007, representing an average annual increase of 0.72 $\mu g/m^{3}$. From 2008 to 2016, there was a relatively stable trend, with the annual average anthropogenic PM_2.5_ concentrations decreasing from 21.89 $\mu g/m^{3}$ in 2008 to 20.15 $\mu g/m^{3}$ in 2016, representing an average annual decrease of 0.19 $\mu g/m^{3}$ (Figure 4). Whereas PM_2.5_ concentration variation was experienced nationally, it was mostly concentrated in a few regions, showing remarkable regional variation. The annual average anthropogenic PM_2.5_ concentrations in eastern and central China showed a similar variation in anthropogenic PM_2.5_. Specifically, the annual average anthropogenic PM_2.5_ concentrations increased from 30.37 $\mu g/m^{3}$ and $19.61\;\mu g/m^{3}$, respectively, in 1998 to 52.38 $\mu g/m^{3}$ and 28.56 $\;\mu g/m^{3}$, respectively, in 2007, representing an average annual increase of 2.2 $\mu g/m^{3}$ and 0.90 $\mu g/m^{3}$ in eastern and central China, respectively. From 2008 to 2016, there was a relatively stable trend, with the annual average anthropogenic PM_2.5_ concentrations decreasing from 50.44 $\mu g/m^{3}$ and 27.71 $\mu g/m^{3}$, respectively, in 2008 to 45.10 $\mu g/m^{3}$ and 25.41 $\mu g/m^{3}$, respectively, in 2016, representing an average annual decrease of 0.59 $\mu g/m^{3}$ and 0.26 $\mu g/m^{3}$ in these regions, respectively. However, the annual average anthropogenic PM_2.5_ concentrations remained stable in western China during the whole period. The annual average anthropogenic PM_2.5_ concentrations increased from 9.87 $\mu g/m^{3}$ in 1998 to 12.71 $\mu g/m^{3}$ in 2016, representing an average annual increase of 0.15 $\mu g/m^{3}$.

Furthermore, in order to quantify the annual anthropogenic PM_2.5_ concentration variations of all gradients in all of China and the three regions from 1998 to 2016, the proportion of land areas of each concentration range was calculated as shown in Figure 4. According to the Chinese ambient air quality standard (GB 3095-2012), the proportion of gradient 1 (≤15 μg/m^3^) significantly decreased from 63.30 to 55.09% from 1998 to 2016 in China, whereas the proportion of gradient 3 (≥35 $\mu g/m^{3}$) significantly increased from 11.34% in 1998 to 16.88% in 2016 in China. Most of the areas of change are mainly located in eastern and central China. For the polarization phenomenon of anthropogenic PM_2.5_ concentrations existing in eastern and central China, the proportion of gradient 1 gradually decreased, and gradient 3 gradually increased. The proportion of gradient 2 remained stable.

### 3.2. Trend Variation in Anthropogenic PM_2.5_ Concentrations

**Figure 5:**
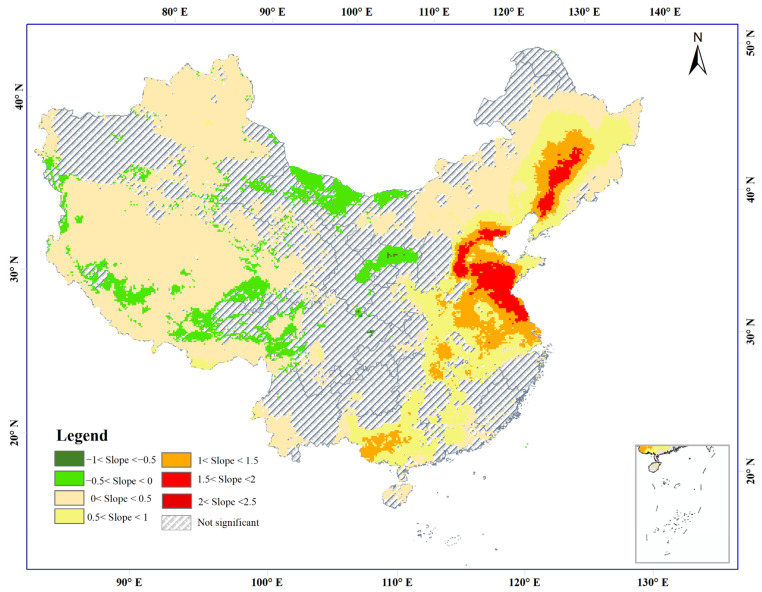
Significance of anthropogenic PM_2.5_ concentrations in China during 1998–2016.

Sen’s slope estimator and the Mann–Kendall test were used to identify trends for anthropogenic PM_2.5_ concentrations in China from 1998 to 2016. The spatial distribution of the variation trend of anthropogenic PM_2.5_ concentrations is heterogeneous in China. The area which experienced a significant increase in anthropogenic PM_2.5_ concentrations between 1998 and 2016 accounts for 54.61% of the total area (trend > 0) and is distributed in the northeast, north, and south-central of China, the Qinghai-Tibet Plateau, and the Xinjiang Autonomous Region (Figure 5). The area which experienced a severe increase in anthropogenic PM_2.5_ concentrations (trend > 1) accounted for 14.33% of the total area and is mainly distributed in the HC, BTH, Shandong and Henan provinces, YRD, and PRD. High anthropogenic PM_2.5_ polluted areas are adjacent to other highly polluted areas. In contrast, the area which experienced a significant decrease in anthropogenic PM_2.5_ concentrations accounts for 6.19% (594,240 km^2^) of the total area (trend < 0) and is mainly concentrated in the Shaanxi and Shanxi provinces.

### 3.3. Impact of Socioeconomic Factors on Anthropogenic PM_2.5_ Pollution

**Figure 6:**
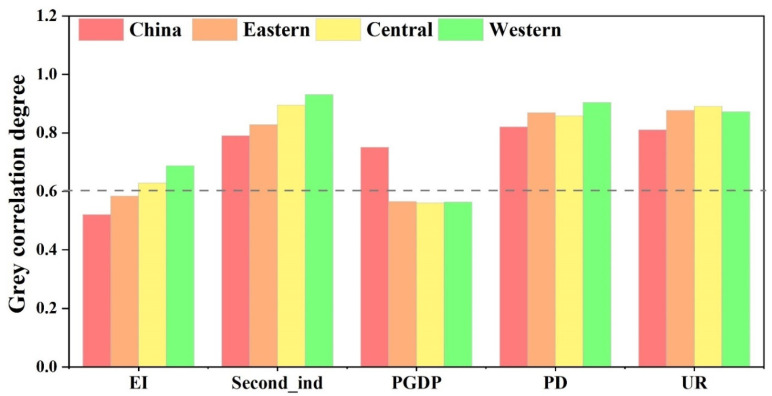
The grey correlation degree between different impact factors and anthropogenic PM_2.5_ concentrations in eastern, central, and western China.

The grey correlation degree between the anthropogenic PM_2.5_ concentrations and impact factors (EI, SI, PGDP, PD, and UR) is calculated according to Table 2. As shown in Figure 6, the grey correlation values between the anthropogenic PM_2.5_ concentrations and impact factors are larger at a moderate or strong level on a national scale. PD and UR have the greater grey correlation value with anthropogenic PM_2.5_ concentrations (0.82, 0.81, respectively). On a regional scale, both similarities and differences in the correlation value among regions and at a national level were observed. In the eastern region, UR has a greater grey correlation value with anthropogenic PM_2.5_ concentrations. In the central and western regions, SI has the greater grey correlation value with anthropogenic PM_2.5_ concentrations.

## 4. Discussion

### 4.1. Trend Analysis of Anthropogenic PM_2.5_ Concentrations

Using Sen’s slope estimator and the Mann–Kendall trend model, it was found that the areas which experienced a significant decrease in anthropogenic PM_2.5_ concentrations were mainly concentrated in the Shaanxi and Shanxi provinces (Figure 5). This finding may be due to a reduction in energy consumption intensity in the Shaanxi and Shanxi provinces. The energy consumption intensity of Shaanxi province dropped from 2.07 in 1998 to 0.62 in 2016, a 70% drop [60]. The energy consumption intensity of Shaanxi province’s drop rate is 2.2 times that of the western region (32%) for 1998–2016. The energy consumption intensity of Shanxi province dropped from 4.07 to 1.5, a 63% drop [60]. The energy consumption intensity of Shanxi province’s drop rate is twice that of the central region (32%) for 1998–2016 [61]. In contrast, more than half of China’s territorial areas of anthropogenic PM_2.5_ concentrations have shown a significant increase, which is mainly distributed in the Ha-Chang-Cheng, Beijing-Tianjin-Hebei, Yangtze River Delta, Changsha-Zhuzhou-Xiangtan, and Pearl River Delta urban agglomerations. These results are consistent with those of other studies and suggest that the anthropogenic activities profoundly affected air pollution when intensive urban development accompanied rapid economic advancement [26,27,62]. With the acceleration of urbanization, industries and populations are concentrated in urban agglomerations [63]. The dust caused by the urbanization process, the exhaust emissions caused by the increase in automobiles, energy consumption, and coal burning for heating, and the burning of straw caused by agricultural production emit a large number of air pollutants [58]. This may be the cause of the significant increase in pollution in the urban agglomerations.

### 4.2. Anthropogenic Effects Were Overshadowed by All Components’ PM_2.5_ Concentrations

Previous studies on all components’ PM_2.5_ concentrations have confirmed that the PM_2.5_ concentrations in the northern region [26,27], especially the desert area of the Xinjiang Autonomous Region, is one of the most heavily polluted areas in China [36,41,64]. We have shown that there are obvious differences in the spatial distribution between anthropogenic PM_2.5_ concentrations and all components’ PM_2.5_ concentrations, especially in the Taklimakan region of the Xinjiang Autonomous Region. Our research results show that the anthropogenic PM_2.5_ concentrations were not in a high-value area in the northern region, especially in the Taklimakan region of the Xinjiang Autonomous Region, which is different from the results of the full-component PM_2.5_ concentrations. This may be due to a combination of natural conditions and human activities. The northern region was located in an arid region, with precipitation less than 200 millimeters, and many areas (Gansu, Qinghai, and Xinjiang) have even less than 50 millimeters [65,66], with low vegetation coverage, bare land cover, and strong winds [67,68]. The characteristics of less precipitation and bare and dry surfaces easily cause sandstorms, and the intensity of sandstorms gradually increases with the increase in surface temperatures [69]. Such a geographic location and climate conditions affect the intensity and spatial distribution of anthropogenic and full-component PM_2.5_ concentrations in the northern region. In addition, human activities in the western region are weaker than those in the central and eastern regions [49]. The urbanization level of the northern region is 20% lower than the urbanization level of the eastern region [70]. In terms of population density, the western region is only 1/3 of the urbanization intensity of the eastern region. This low urbanization rate and low population density lead to low anthropogenic PM_2.5_ concentrations in the western region. We analyzed temporal and spatial variation anthropogenic sources of PM_2.5_, identified the difference between the anthropogenic sources and all-component PM_2.5_ concentrations and further emphasized the importance of identifying anthropogenic and natural sources. Although natural sources also pose health risks for humans [71], pollution control strategies would be inherently different for natural versus anthropogenic sources of PM_2.5_ [72], and the toxicities of natural and anthropogenic sources of PM_2.5_ are not identical due to the distinctive chemical compositions varying between these two forms of PM_2.5_ concentrations. Additionally, ignoring the difference between natural and anthropogenic sources regarding the pathogenic mechanism of PM_2.5_ concentrations will also lead to bias in assessing the health risks and impacts [18]. In addition, there are differences in the formation mechanism of natural and anthropogenic sources of PM_2.5_ pollution, and different pollution sources have different prevention and control measures. If we only consider all-component PM_2.5_ concentrations, researchers may seriously misguide decision-makers in their efforts to improve environmental conditions and protect human health.

### 4.3. Analysis of the Socioeconomic Drivers of Anthropogenic PM_2.5_ Concentrations

In this section, we will elaborate on the driving forces for anthropogenic PM_2.5_ pollution in areas of China where such pollution significantly increased between 1998 and 2016. We found a detailed and diverse impact factor for anthropogenic PM_2.5_ concentrations across regions. Some important observations can be made from the aforementioned detailed and diverse driving factors for anthropogenic PM_2.5_ concentrations across regions. Our findings show that PD and UR have the highest impact in the whole of China and the three regions. The present findings seem to be consistent with other research. Another possible explanation for this is that the increase in population density increases the energy demand and motor vehicle ownership, leading to a continuous increase in energy consumption and vehicle emissions in the local area. In addition, areas with a high population density are short of land resources and densely built, which can easily cause tidal road congestion and poor urban air circulation, creating conditions for PM_2.5_ pollution [24,37,73,74]. In terms of urbanization levels, driven by the development of urbanization, more regions join in the competition to attract investment, and the construction scale of industrial development is increasing [75]; the natural land has rapidly been converted to construction land for traffic, dwellings, and industry use, which bears most of the human activity [76,77]. During this transition process, the increasing road dust and traffic emissions and the increasing number of construction sites have a negative impact on urban air quality [47,50]. The transformation of natural urban land into impervious surfaces directly leads to a reduction in green space, and a decrease in arable land and the ability of ecosystems to absorb, adsorb, and purify particulate matter [5,10,49,78]. In addition, urbanization also indirectly affects air pollution by influencing the urban climate and heat island effect [2,79,80,81].

Comparing the drivers in the three regions, we found two interesting results. Firstly, the impact of SI on anthropogenic PM_2.5_ pollution in the western and central regions is greater than that in the eastern region. Since the “Reform and Opening-up” policy was established in 1978, China has now formed an economic development pattern centered on the eastern region [5]. The eastern region has become the main carrier of urbanization [82], which gathers more than 90% of the Chinese population and GDP, showing strong economic and social functions [73,83]. The eastern region has formed a service-oriented industrial model with low resource consumption and pollution emissions [49]. This may have led to the fact that SI has less influence on PM_2.5_ concentrations in the east than in the central and western regions. In contrast, the economic development of the western region lags behind that of the central and eastern parts [84]. It has long been an energy supply and industrial base, and a special feature of China’s energy sector that is less influenced by foreign investment and trade [82]. There is limited space for enhancing resource performance through technology exchange and upgrading. The western region is currently facing a pressure situation for economic development, and its fiscal revenue depends mainly on secondary industry (with high pollution and energy consumption). The western region is still staying at the stage of scale wins and low-end expansion [83], and this irrational development approach has led to SI becoming the most influential factor compared to the eastern and central regions.

Secondly, the grey correlation degree of impact of EI on anthropogenic PM_2.5_ concentrations in the western region is greater than in the central and eastern regions. Energy intensity, as a measure of the index of energy efficiency, is closely related to production levels, lifestyle choices, and the technological level of a region [85,86]. The western region has long been an energy supply base [73]. Industrial development in the western region shows high energy consumption and pollution characteristics [87]. In contrast, the eastern region has reached a relatively developed level of modernization and service industry to medium-developed countries [49]. The eastern region has formed a service-oriented industrial model with low resource consumption and pollution emissions [49].

China’s energy consumption intensity presents a significant pattern of “high in the west and low in the east” [88]. The difference in the energy consumption structure and technological level between regions is the main reason for the changes in the pattern of China’s energy consumption intensity on a macro scale [89]. Central and western China have China’s main coal bases, and the proportion of coal in the energy structure has been higher than that in eastern China [90]. The National Bureau of Statistics for China in 2015 showed that Shanxi, Inner Mongolia, Ningxia, and other central and western provinces accounted for more than 70% of coal use, whereas eastern provinces such as Zhejiang, Guangdong, and Shanghai accounted for less than 40% of coal use [91]. In addition, technological innovation is an important driving force for the reduction in EI [92]. The average annual expenditure on research and experimental development projects in the eastern region was CNY 155.76 billion, much higher than that in the central region (CNY 37.82 billion) and the western region (CNY 26.38 billion) from 2001 to 2012. Among the eastern, central, and western regions of China, the western region has the greatest potential to improve its energy efficiency [50,93,94].

### 4.4. Implications of This Study

The above results and discussion have important policy implications. The Chinese government must formulate a differentiated anthropogenic PM_2.5_ concentration governance policy. Due to the different resource conditions, geographical features, and anthropogenic PM_2.5_ pollution in the various regions of China, prevention and control policies for anthropogenic PM_2.5_ concentrations should be tailored to the local conditions. According to each region’s characteristics, corresponding governance policies should be formulated to ensure the effectiveness of governance. To be specific, firstly, ecological construction projects should be launched in the desert regions of northwest and northern China. These could be projects to return farmland to forest and grassland in order to increase vegetation coverage, conserve water sources, and prevent wind erosion and sand movement. Increased vegetation will provide benefits such as air purification, and dry and wet deposition, thus reducing PM_2.5_ pollution levels [10]. Secondly, scientific and technological innovation can effectively change the direction of economic development, leading to the transformation and upgrading of the urban economy as well as helping to reduce resource consumption and improve energy efficiency. Thirdly, strengthening urban landscaping, increasing the per capita green park area of cities, and improved landscaping can all play roles in reducing anthropogenic PM_2.5_ concentrations. Finally, western China contains a large amount of wind and solar energy resources [95]. Therefore, the western region should vigorously develop new energy industries and use clean energy such as natural gas, nuclear power, and renewable energy to replace traditional fossil energy.

### 4.5. Uncertainty Analysis

In this research, we illustrated the spatiotemporal dynamics and drivers of anthropogenic PM_2.5_ pollution in China from 1998 to 2016 comprehensively by combing the Sen’s slope, the Mann–Kendall trend model, and GCA. There are still some uncertainties in this study. First, the anthropogenic PM_2.5_ data (PM_2.5_ removed from dust and sea salt) used in this study are difficult to apply to some specific areas, such as areas where there is a large amount of nonanthropogenic biomass burning (e.g., the Amazon rainforest). However, this factor does not affect our major findings, as PM_2.5_ pollution from nonanthropogenic biomass burning only accounts for a small fraction of PM_2.5_ pollution [28,29]. Secondly, our study was unable to identify the risk factors for anthropogenic and natural dust components. A more refined risk estimation of the association between specific PM_2.5_ concentration components and health outcomes based on good cohort data is an important area for future work to address. These results are consistent with previous studies and suggest that anthropogenic sources profoundly affect air pollution when intensive urban development is accompanied by rapid economic advancement [26,27,62]. The acceleration of urbanization, industrialization, and population growth has been concentrated in urban agglomerations [63]. Activities such as agricultural production, straw burning, vehicle exhaust emissions, coal-fired heating, and dust-producing construction emit a large number of air pollutants [96]. These sources may be the causes of the significant increase in pollution. Finally, when examining the impacts of socioeconomic factors on PM2.5 concentrations and change, this study did not consider the impacts of natural variances, such as variations and changes in meteorological conditions (rainfall, wind speed, and direction). Different meteorological factors such as wind speed, relative humidity, and temperature were tightly correlated with PM_2.5_ pollution in urban areas. In the future, it is necessary to comprehensively consider the combined effects of socioeconomic factors and natural factors (terrain and meteorological factors).

## 5. Conclusions

This study quantitatively assessed the temporal and spatial patterns of anthropogenic PM_2.5_ pollution in China. Taking advantage of the high-resolution complete coverage anthropogenic PM_2.5_ concentrations driven by satellite data, we had the opportunity to detect local spatial distribution and temporal variations in the anthropogenic PM_2.5_ concentrations and historical anthropogenic PM_2.5_ concentration trends. A better understanding of the spatiotemporal patterns of anthropogenic PM_2.5_ concentrations and impact factors benefits policymakers tasked with formulating air pollution mitigation strategies. In this study, we found that (1) the average annual anthropogenic concentration of PM_2.5_ in China increased quickly and reached peak value in 2007 with concentrations remaining stable in subsequent years; (2) only 63.30 to 55.09% of the land area in China has PM_2.5_ concentrations that have reached the threshold value of 15 $\mu g/m^{3}$; (3) regarding the polarization phenomenon of anthropogenic PM_2.5_ concentrations existing in eastern and central China, the proportion of gradient 1 (≤15 μg/m^3^) gradually decreased, and gradient 3 (≥35 $\mu g/m^{3}$) gradually increased; and (4) UR, PD, and SI were the main socioeconomic factors affecting the formation of anthropogenic PM_2.5_ concentrations in eastern, central, western China, independently. The increase in EI has a greater potential for mitigating anthropogenic PM_2.5_ emissions in central and western China. These findings allow an interpretation of the spatial distribution of anthropogenic PM_2.5_ concentrations and the mechanisms influencing anthropogenic PM_2.5_ concentrations, which can help the Chinese government develop mitigation PM_2.5_ pollution abatement strategies [97,98,99,100].

## Figures and Tables

**Table 1:** Socioeconomic factors analyzed as potential driving force factors for the spatial variation in anthropogenic PM_2.5_ concentrations.

Variable	Symbol	Unit
Urbanization	UR	%
Population density	PD	people/km^2^
Per capita GDP	PGDP	CNY/km^2^
Energy intensity per GDP	EI	Tons of standard coal/CNY 10,000
Proportion of secondary industry to GDP	SI	%

**Table 2:** Summary statistics of the panel data.

Panel	Statistic	Anthropogenic PM_2.5_ Concentration	UR	PD	PGDP	EI	SI
China	Mean	19.86	0.46	137.60	0.64	1.07	0.46
	St. Dev	2.43	0.08	4.25	0.02	0.35	0.02
	Min	15.10	0.34	130.16	0.61	0.54	0.40
	Max	22.34	0.57	144.25	0.68	1.54	0.49
Western	Mean	12.51	0.40	79.20	1.68	1.45	0.44
	St. Dev	1.29	0.06	1.37	1.22	0.49	0.04
	Min	9.61	0.31	76.67	0.42	0.73	0.39
	Max	14.15	0.51	82.19	3.92	2.24	0.50
Central	Mean	25.08	0.45	150.33	1.99	1.33	0.47
	St. Dev	3.43	0.06	3.75	1.38	0.47	0.04
	Min	18.74	0.36	145.37	0.50	0.65	0.41
	Max	30.37	0.55	156.72	4.38	1.99	0.53
Eastern	Mean	44.43	0.61	498.60	3.67	0.97	0.45
	St. Dev	6.73	0.06	35.20	2.27	0.36	0.02
	Min	30.37	0.51	444.82	1.02	0.39	0.39
	Max	52.38	0.69	546.82	7.62	1.53	0.48

## Data Availability

Data, models, or code generated used during the study are available from the corresponding author by request.
